# Innate Immune Cell Death in Neuroinflammation and Alzheimer’s Disease

**DOI:** 10.3390/cells11121885

**Published:** 2022-06-10

**Authors:** Yetirajam Rajesh, Thirumala-Devi Kanneganti

**Affiliations:** Department of Immunology, St. Jude Children’s Research Hospital, Memphis, TN 38105, USA; rajesh.yetirajam@stjude.org

**Keywords:** neuroinflammation, innate immunity, cell death, pyroptosis, apoptosis, necroptosis, inflammasome, caspase-1, caspase-3, caspase-6, caspase-7, caspase-8, caspase-9, RIPK1, RIPK3, ZBP1, NLRP3, AIM2, RIPK1, MLKL, Toll-like receptor, PANoptosis, PANoptosome, Alzheimer’s disease, Amyloid β, Tau, microglia

## Abstract

Alzheimer’s disease (AD) is a neurodegenerative disorder molecularly characterized by the formation of amyloid β (Aβ) plaques and type 2 microtubule-associated protein (Tau) abnormalities. Multiple studies have shown that many of the brain’s immunological cells, specifically microglia and astrocytes, are involved in AD pathogenesis. Cells of the innate immune system play an essential role in eliminating pathogens but also regulate brain homeostasis and AD. When activated, innate immune cells can cause programmed cell death through multiple pathways, including pyroptosis, apoptosis, necroptosis, and PANoptosis. The cell death often results in the release of proinflammatory cytokines that propagate the innate immune response and can eliminate Aβ plaques and aggregated Tau proteins. However, chronic neuroinflammation, which can result from cell death, has been linked to neurodegenerative diseases and can worsen AD. Therefore, the innate immune response must be tightly balanced to appropriately clear these AD-related structural abnormalities without inducing chronic neuroinflammation. In this review, we discuss neuroinflammation, innate immune responses, inflammatory cell death pathways, and cytokine secretion as they relate to AD. Therapeutic strategies targeting these innate immune cell death mechanisms will be critical to consider for future preventive or palliative treatments for AD.

## 1. Introduction

Alzheimer’s disease (AD) is a debilitating disease affecting approximately 44 million people worldwide [1]. The progression of this neurodegenerative disease varies between patients, and symptoms can take decades to develop. Dementia, characterized by progressive memory loss and cognitive impairment, is most often caused by AD. As dementia worsens, patients become unable to care for themselves and need constant monitoring and assistance before eventually succumbing to the effects of the disease. For these reasons, AD places an enormous burden on both individuals and society [2].

AD is characterized by the presence of two structural brain abnormalities caused by the proteins amyloid β (Aβ) and type 2 microtubule-associated protein (Tau). The characteristic AD-inducing plaques, caused by Aβ deposition between nerve cells in the brain, can manifest several years before clinical symptoms unfold, and plaques play a major role in cognitive decline and neuronal cell death [3,4]. Aβ is formed from amyloid precursor protein (APP) cleavage; APP is expressed in neurons and glial cells and is responsible for mediating cell-to-cell adhesion, neuronal signaling, and regulating neurotransmitter discharge [5]. APP can be cleaved into peptides (37–49 amino acids in length) by several proteolytic secretases. Depending on which secretases cleave APP, the process is either termed amyloidogenic, which is associated with plaque formation and disease, or nonamyloidogenic [6]. The nonamyloidogenic pathway is initiated by APP cleavage by α-secretase, followed by γ-secretase. The product is a non-toxic, soluble APPα fragment with essential neuroprotective functions [6,7]. Alternatively, induction of the amyloidogenic pathway involves APP cleavage by β-secretase, which first generates a soluble APPβ peptide. This processing is followed with cleavage by γ-secretase, which cleaves the APP C-terminal fragment, generating neurotoxic Aβ peptides. Various lengths of Aβs form, but Aβ_1-42_ is the major component of plaque formation; Aβ_1-42_ has a highly hydrophobic C-terminus that initiates Aβ aggregation and oligomerizes into higher order insoluble structures that diffuse throughout the brain [6,7,8]. These plaques interrupt cellular communication and can cause microglial activation and inflammation that ultimately can cause neuronal death and tissue damage in the brain [9].

Along with Aβ aggregation, the dysregulation of the protein Tau also results in AD [2]. Tau is expressed in neurons, astrocytes, and oligodendrocytes. Normally, Tau binds microtubules to stabilize them, impacting synaptic functions [10]. However, abnormal post-translational modifications can hyper-phosphorylate Tau, causing dissociation from microtubules and intracellular aggregation via neurofibrillary tangles (NFTs) [11]. Tau hyperphosphorylation and NFTs are associated with AD pathogenesis [12,13]. Additionally, Aβ deposition can drive Tau-mediated AD pathogenesis resulting in NFTs, cognitive impairment, and dementia [2].

While Aβ plaques and NFTs in neurons are key features of AD, the disease pathogenesis is not limited to the neuronal compartment, and many of the brain’s immunological cells (astrocytes, microglia, and peripheral infiltrating immune cells) are also involved. The activation of astrocytes and microglial cells releases proinflammatory cytokines, including IL-1β, TNF-α, and IL-6; these cytokines can trigger Tau hyper-phosphorylation [2]. Additionally, increased levels of proinflammatory cytokines have been found in the serum and brains of patients with AD compared with healthy patients, and this prolonged neuroinflammation can lead to the misfolding of Tau [14]. These results collectively lay the groundwork for a strong connection between innate immunity and pathology in the central nervous system (CNS) [15,16].

### Innate Immune Signaling Pathways in the Central Nervous System

Innate immune signaling pathways in microglial cells are activated in response to a myriad of stimuli such as infection, injury, and chronic disease. As a result of an activated immune response, programmed cell death (PCD) can occur and has a significant role in pathogenesis in the CNS, with specific roles in brain development, homeostasis, and clearance of infected/transformed cells. There are several forms of PCD with defined molecular signatures [17], ranging from the traditionally noninflammatory apoptosis pathway to highly proinflammatory forms of cell death such as pyroptosis and necroptosis. Additionally, growing evidence of crosstalk among these three pathways has led to the conceptualization of PANoptosis, an inflammatory cell death pathway that integrates components from other cell death pathways. The totality of biological effects in PANoptosis cannot be individually accounted for by pyroptosis, apoptosis, or necroptosis alone [18,19,20,21,22,23,24,25,26,27,28,29,30,31,32,33,34,35,36]. Historically, apoptosis was considered to be the primary form of PCD employed in the CNS [37]. Apoptosis is initiated by microenvironmental perturbations that result in initiator caspases (caspase-8 or -9) activating downstream executioner caspases (caspase-3, -6, and -7) [17]. In neurological diseases, markers of apoptotic cell death have been identified, including the presence of pro-apoptotic B-cell lymphoma-2 (Bcl-2) family members, the activation of caspases, and the cleavage of caspase substrates [4]. However, nonapoptotic inflammatory PCD pathways also play a significant role in neurodegeneration. Pyroptosis is a proinflammatory, lytic PCD pathway that is associated with pathology in AD, multiple sclerosis, and traumatic brain injury [38]. Canonical pyroptosis is a caspase-1–mediated cell death pathway that involves inflammasome formation and the activation and release of proinflammatory cytokines IL-1β and IL-18 [38,39,40]. Necroptosis, another lytic form of PCD that occurs in response to caspase-8 inhibition and is RIPK3- and MLKL-dependent [17,41,42,43], has also been shown to be involved in the pathophysiology of neurological diseases such as ALS, schizophrenia, and ischemia–reperfusion injury [44,45,46]. The activation of RIPK1, a key inflammatory PCD molecule, may also lead to neuroinflammation and cell death [45].

Many questions remain about the precise mechanisms through which innate immunity and PCD pathways modulate neuronal pathogenesis. Here, we discuss the link between neuroinflammation, innate immune responses, and AD. Innate immune sensors and molecular mechanisms of PCD, as related to AD, are also described, along with proposed AD therapeutic strategies targeting fundamental regulators of PCD. Considering the innate immune components of AD pathogenesis and identifying strategies to therapeutically target these pathways will be critical for informing clinical interventions.

## 2. Neuroinflammation, Innate Immunity, and AD: A Complex Relationship

Neuroinflammation is involved in the propagation of several neurodegenerative disorders and has been shown to be a major contributor to AD pathogenesis and progression. Inflammation in the CNS can occur as a result of cells sensing Aβ or other damage- or pathogen-associated molecular patterns (DAMPs or PAMPs). Cells contain several pattern recognition receptors (PRRs), both on the cell surface and in the cytoplasm, that are responsible for recognizing DAMPs and PAMPs; sensing can induce inflammatory signaling pathways and immune responses. The five primary PRR families include Toll-like receptors (TLRs), retinoic acid-inducible gene-I (RIG-I)-like receptors (RLRs), nucleotide-binding oligomerization domain (NOD)-like receptors (NLRs), C-type lectin receptors (CLRs), and the absent in melanoma 2 (AIM2)-like receptors (ALRs) [47]. This PAMP/DAMP-mediated signaling response incites the production of inflammatory cytokines and chemokines, the induction of cell death to clear the infected cells, and in the case of AD, Tau protein misfolding or hyperphosphorylation and increased Aβ pathology [2,14]. Inflammatory responses in the CNS are largely carried out by glial cells, including microglial cells and astrocytes, though endothelial cells also play a role. Below, we highlight the key molecular pathways involved in DAMP and PAMP sensing and cytokine release in these cell types.

### 2.1. Microglia

Microglia are self-renewing immune cells of the CNS [48] that arise from yolk-sac fetal macrophages [49,50,51]. They play an essential role in regulating brain homeostasis and facilitating immune responses [52,53]. Microglial cells patrol the environment, assess and maintain synaptic health, clear debris, and assist in neuronal survival [54]. In response to stimuli, microglia can undergo classical activation to develop towards an M1 phenotype, which promotes inflammation, or they can undergo alternative/neuroprotective activation and develop towards an M2 phenotype, which is associated with anti-inflammatory functions. Healthy CNS maintenance requires a balance of M1 and M2 microglial activation to repair tissues, an M2 function, and also to clear cellular debris and aggregated misfolded proteins, an M1 function [55,56]. While these functions are essential for homeostasis, the overactivation of these pathways can lead to pathology.

Approximately 25% of the ~84 risk genes in AD that are associated with immune function have been identified to be enriched or exclusively expressed in microglia [57]. Some of these genes are *TREM2, CD33, INPP5D, CLU, CR1, SPI1, ABCA7, EPHA7, MS4As, HLA-DRB5-DRB1, CASP7,* and *CASP8* (Table 1). Further research is needed to understand how these specific risk variants impact protein function, which could aid in identifying therapeutic targets for AD treatment [57]. In addition to these gene associations, several molecular mechanisms for innate immune activation in microglial cells have been implicated in AD pathogenesis. Microglia develop toward the proinflammatory M1 phenotype after sensing DAMPs and PAMPs through PRRs that include TLRs, RLRs, and NLRs [9,58,59,60,61]. In neurodegenerative diseases such as AD, PRRs are highly expressed in microglia; signaling through PRRs can provoke an inflammatory response and proinflammatory cytokine secretion [62,63] (Table 2). In healthy controls, activated microglia localize around Aβ plaques and neurons with NFTs [64,65]. Microglia use cell surface receptors (CD14, TLR2, TLR4, α6β1 integrin, CD47) and scavenger receptors (CD36) to phagocytose and subsequently clear Aβ [66,67,68,69]; specifically, the physical interaction of TLR2, TLR4, and the TLR4-coreceptor CD14 activates an immune response to fibrillar Aβ phagocytosis [70]. While microglial activation is beneficial in preventing AD-associated pathology, the chronic activation of microglia is detrimental, as prolonged TLR2 and TLR4 activation in microglia induces Aβ production [71]. If Aβ plaque formation is extensive, microglial cells cannot eliminate it [72,73,74,75]. In a common AD mouse model where mice express the double transgenic APP/PS1 (chimeric mouse or human APP/human presenilin 1 [76]), the inhibition of TLR2 reduces glial cell reactivity, reduces Aβ, and improves cognitive function [77]. Furthermore, TLR2/4-deficient C57BL/6 mice exhibit improved neuro-cognitive and behavioral patterns compared to wild-type mice in response to the Aβ_1–42_ peptide [78], and TLR2-deficient microglia induce proinflammatory cytokine release (TNF-α, IL-1β, IL-6) and enrich Aβ clearance [79,80]. These results highlight the importance of TLRs in driving AD pathology.

Other PRRs, such as inflammasome sensors, have also been associated with AD. Inflammasomes are multiprotein complexes that form in response to PAMP or DAMP sensing, and they generally contain a sensor, the adaptor protein ASC, and caspase-1. Several inflammasome sensors have been described, with the most well-characterized being the NLR family sensors, NLRP1 [40], NLRP3 [95,96,97], and NAIP/NLRC4 [98,99,100], as well as other sensors containing pyrin domains, such as Pyrin [101] and AIM2 [102,103]. The NLRP3 inflammasome, which has been implicated in autoinflammatory diseases, obesity, colitis, cancers, and infections [104], has emerged as a trigger of AD pathology. Tau can activate the NLRP3 inflammasome in microglia, inducing Tau pathology and potentiating AD pathogenesis [105]. Similarly, NLRP3-deficient APP/PS1 mice are protected against neurobehavioral deficits and have reduced spatial memory deficits and Aβ buildup [88].

In addition to the direct role of PRRs in AD pathogenesis, PRR activation and signaling in microglia can also induce the release of proinflammatory cytokines that drive pathology. Inflammation or injury to the neuron can activate microglia to generate proinflammatory factors (IL-1β, TNF-α, and IL-6; an M1 phenotype). Nucleic acid-containing Aβ elicits a TLR- and RLR-derived type I IFN response for C3-dependent synapse destruction [106]. The expression of RIG-I, a key member of the RLR family, is elevated in the temporal cortex and plasma of patients with cognitive impairment [94]. However, the mechanistic details of RIG-I involvement in AD pathogenesis have yet to be discovered.

The activation of PRRs, especially inflammasome sensors, can lead to the release of IL-1β and IL-18, and these proinflammatory cytokines are also correlated with AD severity [107,108,109,110]. Though the release of proinflammatory cytokines is a natural result of the aging process, their continuous release can lead to Aβ production and neuronal distress [111,112,113,114,115,116].

### 2.2. Astrocytes

The CNS relies heavily on astrocytes for maintaining homeostasis and assisting with synapse formation and elimination [117,118]. Astrocytes are more numerous than neurons [119] and have a significant role in activation, neuroprotection, and neurotoxicity [120,121]. They respond to cytokines/chemokines and can identify Aβ aggregates [118,122,123], undergo hypertrophy upon activation, and upregulate glial fibrillary acidic protein (GFAP) expression [124]. Reactive astrocytes (atrophied or disrupted intralaminar astrocytes) are characteristically present in AD brains [125]. While innate immune sensing in astrocytes is not well understood, inflammasome activation in astrocytes has been associated with some neurodegenerative diseases. The AIM2 inflammasome was found to be activated during experimental autoimmune encephalomyelitis in astrocytes, although these cells failed to undergo cell death and had poor IL-1β expression [126]. Additionally, astroglial NLRP3 inflammasome complexes have also been reported to be involved in neuroinflammation in ALS, with higher levels of NLRP3, ASC, IL-18, and caspase-1 in comparison to non-diseased controls [127]. The role of innate immunity in astrocytes during AD requires further characterization.

### 2.3. Endothelial Cells

Single-nucleus transcriptome analyses from prefrontal cortical samples from patients with AD identified a variety of endothelial transcriptomic changes, specifically in angiogenesis and the immune response pathway when compared with healthy patients [128]. The endothelial cells of AD patients showed an elevated expression of *EGFL7, FLT1,* and *VWF* (angiogenic growth factors and their receptors) and *B2M* and *HLA-E* (antigen presentation machinery) [128], suggesting that these cells play a role in angiogenesis and immune responses in a diseased state. However, this cell type remains largely under-characterized for its roles in AD.

## 3. Cell Death and AD

The major pathological hallmarks of AD include extracellular Aβ deposition, the intraneuronal aggregation of NFTs, and neuronal loss (neurodegeneration) [129]. In mammalian hosts, Aβ and Tau deposits act as DAMPs and are recognized by multiple PRRs, as discussed above [68,88,94,130,131] (Table 2). The subsequent innate immune signaling can result in cell death, and these processes can provide significant protective responses against AD [62,88,131]. Conversely, inflammatory cell death also releases proinflammatory cytokines and cellular contents that can stimulate severe inflammation [132,133]. There is a delicate balance between an appropriate immune response, which clears Aβ and Tau deposits, and an exaggerated response, which promotes neuroinflammatory brain damage [132,133]. Below, we discuss the mechanics of canonical PCD pathways and what is known about their involvement in AD.

### 3.1. Pyroptosis in AD

Pyroptosis is an inflammatory PCD pathway initiated by the assembly of a multimeric protein complex called the inflammasome [40], as described above. The canonical inflammasome assembly cleaves pro–caspase-1, allowing its active form to cleave downstream substrates. These substrates include gasdermin D (GSDMD), leading to the release of its N-terminal fragment to form pores in the plasma membrane, and pro–IL-1β and pro–IL-18, which are processed into their active forms for release through the membrane pores [134,135,136,137,138]. Since activated caspase-1 mediates potent cell death and an inflammatory response, its catalytic activity is under tight regulation [139,140].

In pyroptosis, caspase-1-dependent GSDMD cleavage, and the subsequent release of IL-1β and IL-18, have been well characterized in immune cells such as macrophages [141]. However, pyroptosis and inflammasome activation also occur in brain cells; activated caspase-1 is elevated in AD brains and in the APP/PS1 mouse model [142], and Aβ_1-42_ can induce pyroptosis in cortical neurons [142,143] (Table 3). Additionally, Aβ fibrils can stimulate NLRP3 inflammasomes via lysosomal damage in mouse microglia [144]. NLRP3 or caspase-1 deficiency in the APP/PS1 mouse model reduces spatial memory impairment, hippocampal synaptic plasticity loss, behavioral disturbances, and AD consequences [88]. NLRP3 inflammasome deficiency also skews microglial cells from the M1 phenotype to the M2 phenotype [88]. Furthermore, microglial cells have been shown to secrete IL-1β after NLRP3 activation with recombinant Tau protein [105,130]. Together, these studies implicate pyroptosis in AD pathogenesis.

### 3.2. Apoptosis in AD

Apoptosis occurs through either the extrinsic or intrinsic pathway. Extrinsic apoptosis is activated by extracellular stimuli through the formation of a death-inducing signaling complex (DISC) that recruits adaptor proteins (including TRADD and FADD) and pro-caspase-8 [155,156]. Caspase-8 can then activate effector caspases, caspase-3, -6 and -7, to drive further substrate cleavage and the execution of apoptotic cell death [157,158]. Intrinsic apoptosis responds to intracellular changes in equilibrium and involves the activation of Bcl-2 family members followed by the release of mitochondrial proteins such as cytochrome c. Cytochrome c associates with APAF-1 and activates caspase-9, which enables downstream effectors, caspase-3, -6, and -7 [157,158,159,160].

Many studies have shown that compared to healthy brains, the expression profiles of several components of apoptosis, most notably the Bcl-2 family members, are altered in AD [161]. Bcl-2 family members can be classified as either pro- or anti-apoptotic, with Bax, Bak, BAD, Bid, Bim, and Bcl-x being canonically pro-apoptotic and Bcl-2, Bcl-xL, and Mcl-1 being anti-apoptotic [161]. Pro-apoptotic Bim is upregulated, anti-apoptotic Bcl-2 is downregulated, and pro-apoptotic Bax is activated in response to Aβ [162,163]. Furthermore, anti-apoptotic Bcl-xL expressed in microglia has been shown to co-localize with Aβ deposits and activate astrocytes [164]. Additionally, Bcl-2 proteins control intracellular calcium, and irregularities in calcium signaling have been implicated in AD progression [161], providing an additional link between apoptotic Bcl-2 proteins and AD.

In addition to the Bcl-2 family members, apoptotic caspases are also associated with AD. Stimulating microglia with inflammogens can activate caspase-8, -3, and -7 in BV2 cells and in mice, and these caspases are activated in the microglia of patients with AD [165]. Molecularly, caspase-3 can activate NF-κB, via protein kinase Cδ, and increase the production of neurotoxic proinflammatory mediators (IL-1β, TNF-α, and NO). Subsequently, in vitro inhibition of caspase-8 impedes microglia activation and neurotoxicity [166]. Caspase-3 can cleave APP, and this cleavage event can provoke Aβ plaque formation and synaptic loss in the brain as well as cause noticeable changes in behavior [147]. Along with caspase-3 activation, activating caspase-8 and -9 also induces Aβ plaque formation [151,153,167,168]. From a therapeutic standpoint, the pan-caspase inhibitor (Q-VD-OPh) and microglial activation inhibitor (minocycline) have been shown to provide neuroprotective effects in TgCRND8 and APP/PS1 mouse models, respectively [169,170] (Table 3). Collectively, these studies emphasize the involvement of apoptotic molecules in AD pathogenesis.

### 3.3. Necroptosis in AD

Necroptosis can be initiated in response to multiple signaling pathways, including TLR signaling, death receptor engagement, and IFN signaling [171]. The necroptotic cell death pathway is activated when caspase-8 is inhibited; RIPK1 is autophosphorylated and, through its interaction with phosphorylated RIPK3, recruits and phosphorylates MLKL [172,173,174,175,176,177,178]. Oligomerized MLKL translocates to the plasma membrane where it interacts with phospholipids to produce membrane pores [172,179,180].

Necroptosis can impair cognitive function; in the APP/PS1 mouse model, mice expressing constitutively active MLKL performed worse in the Morris water maze and had fewer neurons [154]. In addition, a robust increase in the levels of RIPK1 and MLKL have been seen in AD brains compared to healthy controls [154] (Table 3). Furthermore, inhibiting RIPK1 reduces Aβ deposits, inflammatory cytokines, and cognitive deficits in the APP/PS1 mouse model [181]. There is also a correlation between necroptosis and reduced brain weight, which is a pathological consequence of AD [154]. Additionally, aberrant protein phosphorylation is a well-recognized component of AD pathogenesis [182], and because the phosphorylation of RIPK1, RIPK3, and MLKL regulates necroptosis, it is possible that AD progression may modulate this cell death pathway.

### 3.4. PANoptosis in AD

PCD pathways have long been thought of as segregated pathways with little overlap. However, numerous studies have found significant crosstalk among the components of pyroptosis, apoptosis, and necroptosis. Components of noninflammatory apoptosis can undergo crosstalk with molecules involved in executing lytic, inflammatory PCD pathways [183,184]. For example, caspase-3 can activate GSDME, inducing a gasdermin-dependent cell death program [185], and caspase-8 can directly activate GSDMD in certain conditions [186,187]. The culmination of the observed experimental crosstalk has led to the conceptualization of PANoptosis. PANoptosis is an inflammatory cell death pathway that integrates components from other cell death pathways. The totality of biological effects in PANoptosis cannot be individually accounted for by pyroptosis, apoptosis, or necroptosis alone. PANoptosis is regulated by multifaceted macromolecular complexes termed PANoptosomes [18,19,20,21,22,23,24,25,26,27,28,29,30,31,32,33,34,35,36]. PANoptosis is initiated by PAMP/DAMP sensing through an upstream sensor, followed by assembly of the multi-protein PANoptosome complex. PANoptosis involves the activation of multiple cell death molecules, which can include caspase-1, gasdermins, caspase-8, caspase-3, caspase-7, caspase-6, MLKL, and potentially others [18,19,20,21,22,23,24,25,26,27,28,29,30,31,32,33,34,35,36]. Molecules shown to be involved in PANoptosis, such as AIM2 [35], caspase-8, caspase-1, RIPK3, and MLKL [18,19,20,21,22,23,24,25,26,27,28,29,30,31,32,33,34,35,36], have been implicated in neuroinflammation and neurodegenerative disorders, including AD (Table 1 and Table 3). Additionally, the increased expression of *CASP1*, *CASP3*, *CASP6*, *CASP7*, *CASP8*, and *CASP9* have all been found in the entorhinal cortex of patients with AD with severe dementia [188], suggesting that pyroptosis or apoptosis alone may not account for the full picture of cell death in AD. Furthermore, inflammasome components are now well-established in their ability to drive PANoptotic cell death responses in mice in response to specific stimuli [18,22,30,34,189,190,191]. To date, Z-DNA binding protein 1 (ZBP1)- and AIM2-PANoptosomes have been characterized [18,22,30,35,36], with more PANoptosomes likely to exist. In the context of AD, AIM2 deficiency has been shown to reduce Aβ deposition and microglial activation in the 5xFAD mouse model [90], suggesting that the AIM2-PANoptosome may play a role in AD. PANoptosis is an emerging concept which, when applied to AD, may allow for a more complete understanding of how PCD impacts AD and other neurodegenerative diseases.

## 4. Cytokines and Chemokines as Modulators of Neuroinflammation

**Figure 1:**
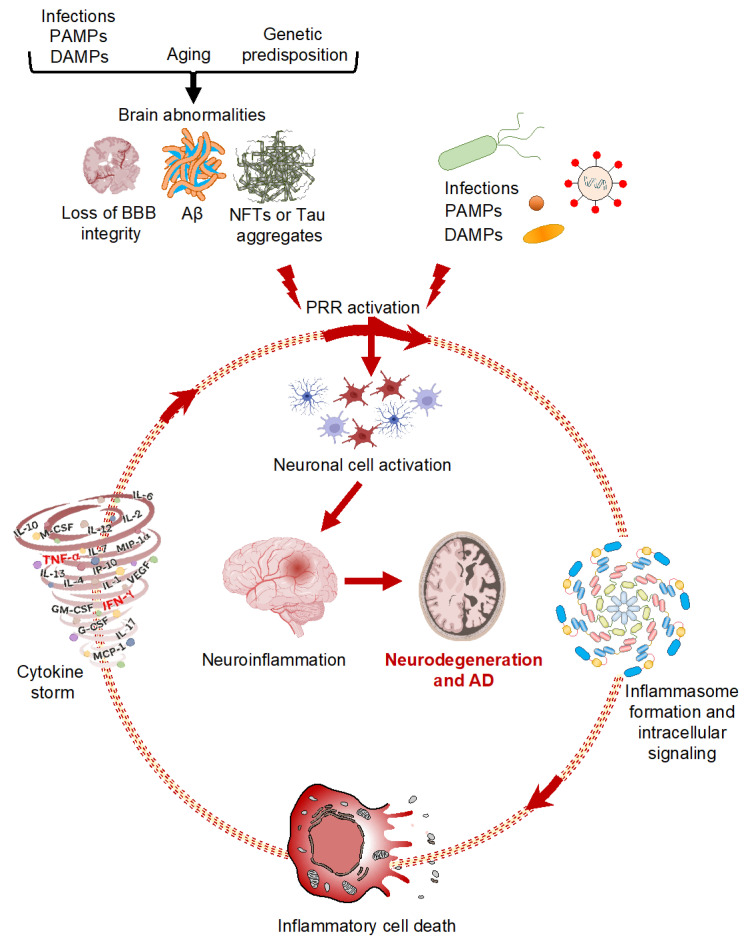
Innate immune signaling and neurodegeneration. Schematic representation of different Alzheimer’s disease triggers activating innate immune signaling, neuroinflammation, and inflammatory cell death in neurodegenerative disorders. Created with Biorender (accessed on 12 May 2022).

Innate immune signaling pathways and cell death mechanisms often culminate in the release of cytokines and chemokines. Cytokines, which are largely released by microglia and astrocytes, are major contributors of neuroinflammation. They are involved in chemoattraction, pro- and anti-inflammatory processes, neuronal injury, and the microglial response to Aβ deposits. Since cytokines modulate microglial activation, their presence or absence can be influential in the development and progression of AD [9] (Table 4). Several disease conditions can release cytokines into the brain; this has been associated with dementia and the loss of neurocognition. For example, associations between dementia and infectious diseases have been reported [192]. Additionally, high levels of intracranial proinflammatory cytokines have been reported during viral infections, including the neuroinvasive SARS-CoV-2 [193,194], suggesting that these infections may also exacerbate neuroinflammation and promote disease. Recent evidence has also identified a mechanistic link between cytokine storms, particularly synergism between TNF-α and IFN-γ, and PANoptotic cell death [31], defining cytokine storms as a life-threatening condition caused by excessive production of cytokines mediated by inflammatory cell death, PANoptosis [195]. Given the established role of TNF-α in AD [78] and the links between cytokine storm and neuroinflammation, this process is likely to further contribute to AD pathology (Figure 1). 

Inflammation must be tightly balanced in the brain. A proinflammatory environment, such as that seen in AD brains, accounts for damaging pathology. The increased caspase-1 activation in AD leads to higher levels of IL-1β release [88,210]; this can be detrimental if prolonged, but there are conditions where a slightly inflammatory environment can be beneficial. For instance, IL-1β expression activates a form of neuroinflammation in the APP/PS1 mouse model that reduces amyloid plaque pathology [211,212], suggesting that a low level of inflammatory cell death could help prevent AD pathology. However, those with mild cognitive impairment are at risk for conversion to AD if they have high levels of the proinflammatory cytokine TNF-α and decreased anti-inflammatory TGF-β in their cerebrospinal fluid (CSF) [213]. Neuronal function is impaired with elevated levels of IL-1β, TNF-α, and other cytokines, as shown by the suppression of long-term potentiation of synaptic transmission [9]. Microglia in AD are skewed toward the M1 phenotype, indicated by a higher expression of proinflammatory cytokines/chemokines and innate immune receptors [9]. Additionally in an AD mouse model (Mo/Hu APPswe PS1dE9 mice), TLR4 mediates higher levels of TNF-α and MIP-1α [214]. Among the proinflammatory cytokines, interferon (IFN) is also a crucial molecule contributing to AD neuropathogenesis; RNA-seq data reported the upregulation of AD-related microglial-specific IFN-stimulated genes (ISGs) that had significant correlation with disease severity and complement activation [106].

In addition to cytokines, chemokines also enhance local inflammation in AD by regulating microglial migration to areas of neuroinflammation [215]. In AD, CCL2, CCR3, and CCR5 are elevated in reactive microglia [216,217], and CCL4 is detected in reactive astrocytes near the vicinity of Aβ plaques [216]. Aβ treatment to human astrocytes and macrophages generates CXCL8 (IL-8), CCL2, CCL3, and CCL4 [218], and microglia produce CXCL8, CCL2, and CCL3 [219]. Additionally, the CX3CR1/CX3CL1 AD mouse model modulates neuronal survival [220], plaque load [221], and cognition [222]. Overall, cytokines and chemokines are important to modulate signaling to help clear DAMPs in the brain, but prolonged production and exposure contributes to pathology and disease in AD.

## 5. Therapeutic Implications of Innate Immune Involvement for AD Management

Several AD therapeutics have attempted to target Aβ plaques and Tau. However, the clearance of Aβ plaques through anti-Aβ immunotherapy has failed to provide any cognitive benefit in patients with AD thus far [223]. The U.S. FDA granted accelerated approval for aducanumab, an anti-Aβ immunotherapy, to be used in patients with AD; however, the drug remains highly controversial with two phase III trials providing conflicting clinical efficacy results and lingering concerns over safety [224,225]. Previous anti-Tau therapies, such as those inhibiting kinases, Tau aggregation, or microtubule stabilization, have been withdrawn from clinical trials because of toxicity and efficacy issues [226]. However, several current Tau-targeted immunotherapies, including AADvac-1, ABBV-8E12, BIIB092, and RO71015705 [227], have shown promising preclinical results and are presently being evaluated in clinical trials [226]; their efficacy in patients with AD remains to be determined. The overall lack of clinical success to date in targeting Aβ plaques and Tau has led to a critical need to identify alternative treatment strategies that target upstream signaling, such as innate immune processes and PCD.

Mouse models, such as the APP/PS1 model as well as others, allow researchers to gain an understanding of relevant disease-related PCD signaling pathways and their regulation. The immediate hope is that PCD, in some capacity, can be targeted to modulate neurological disease severity and progression. However, this approach has also faced challenges. Historically, there has been an association between apoptosis and the expression of key apoptotic proteins with neurodegenerative disorders; yet in vivo targeting of apoptosis has failed to show effective therapeutic results. A recent clinical trial for minocycline, a 2^nd^ generation tetracycline blocking cytochrome c release and upregulating Bcl-2 expression, was ineffective in patients with mild AD [228,229]. This may be due in part to crosstalk between PCD pathways. In contrast, the pharmacological inhibition of RIPK1 to prevent necroptosis results in neuroprotection in preclinical AD models [4], suggesting that targeting cell death remains a potentially viable therapeutic strategy for AD. Therefore, several other clinical trials and preclinical studies involving compounds that target cell death molecules are currently ongoing (Table 5).

A correlation has also been identified between inflammatory cytokines and their signaling pathways and AD, with microglia playing a key role in coupling inflammation with neurodegeneration [88,131,243,244]. Therefore, neuroinflammatory biomarkers are also being evaluated as potential targets (Table 5). However, AD pathogenesis may also be prevented through the adaptive immune system and the regulation of microglial function [245].

In addition to immune, inflammatory, and cell death pathways, the meningeal lymphatic vasculature (mLV) system may also be a promising therapeutic target for AD. The mLV system moves immune cells from the brain to the peripheral immune system; it is crucial in maintaining fluid homeostasis and enabling appropriate innate immune responses [246,247]. Mice with altered mLV have worsening AD symptoms, and the photoablation of mLV in 5xFAD mice leads to increased Aβ deposition, neurovascular dysfunction, and cognitive defects [248]. Dysfunctional mLV in murine models can also induce the activation of CNS immune cells and the increased secretion of proinflammatory cytokines IL-6, TNF-α, and IL-1β [246,249]. These data suggest that mLV, through modulating inflammation, could be involved in the progression of neurodegenerative diseases such as AD and warrants further study to determine whether mLV could be considered as a therapeutic target.

## 6. Discussion and Future Directions

Recent studies have significantly expanded our understanding of neuroinflammation, innate immune responses, and tightly orchestrated cell death signaling in AD pathogenesis. The brain immune cells (microglia) are integrally associated with innate immune cell death in AD pathology. Excessive neuroinflammation during Aβ and Tau clearance could be diminished by specifically regulating the innate immune cell death response. The continued elucidation of cell death pathways and the central innate immune sensor signaling pathways involved in regulating neuroinflammation and Aβ/Tau clearance will have a significant impact on the AD research field. The emerging concept of PANoptosis is likely to play a role in disease pathogenesis and understanding its relevance in AD therapeutics. Emerging AD studies are focusing on this network of inflammasomes, inflammatory cell death, and neuroinflammation. Further investigations into these processes will potentially aid in identifying novel therapeutic targets for AD management.

## Figures and Tables

**Table 1:** Summary of immune genes that have mutations associated with AD.

Gene	Category	Lead Variant	Cell Type	Role in AD Pathogenesis	Reference
***TREM2***	Immune receptor	rs187370608	Microglia	Negatively impacts binding to cell-surface TREM2 ligands and Aβ oligomers	[57,81,82,83,84,85]
		TREM2 R47H		Late onset of AD	
		T96K		Gain-of-function mutation, resulting in increased cellular binding	
***CD33***	Immune receptor	Minor allele of CD33 SNP rs3865444	Microglia	Confers protection against AD; reduced levels of full-length CD33 and insoluble Aβ42	
***INPP5D***	Signaling intermediate	Intron variant rs10933431	Microglia	−	
***CLU***	Complement	Intron Variant rs4236673	Astrocyte	Aβ clearance	
***CR1***	Complement	Intron variant rs2093760	Microglia	Aβ clearance	
***SPI1***	Transcription factor	−	Microglia	Aβ clearance	
***ABCA7***		−	Neuron	Aβ clearance	
			Microglia	APP processing	
***EPHA1***	Effector mechanism	Intron variant rs7810606	Oligodendrocyte	Tau pathology	
			Microglia		
***MS4As***	Immune receptor	Intergenic rs2081545	Microglia	−	
***HLA-DRB5-DRB1***	Immune receptor	Intergenic rs6931277	Microglia receptor	−	
***CASP7***	Cell death	Missense variant Gene wise	−	−	
***CASP8***	Cell death	Missense variant Gene wise	Neurons	Amyloid processing	

**Table 2:** Innate immune molecules involved in Alzheimer’s disease.

Sensors	Model System	Cell Type	Mechanism	Role in AD Pathogenesis	Reference
**TLRs**					
**TLR2**	APP/PS1 mouse model	Microglia	TLR2 deficiency in microglia induces expression of TNF-α, IL-1β, IL-8, and enhanced Aβ clearance	High extracellular Aβ deposits; impairs cognitive function	[77,79,80]
			Inhibition of TLR2 activity attenuates glial cell reactivity and leads to reduction in Aβ deposits		
**TLR4**	APP/PS1 mouse model	Microglia	TLR4 deficiency reduces microglial activation, and its activation in microglia enhances production of Aβ peptides	Alteration in extracellular Aβ deposits	[71,86]
	AD samples		Increased amounts of inflammatory cytokines such as TNF-α, IL-1β, IL-6, and IL-8	Impairs cognitive function	
**TLR9**	Tg2576 mouse model	Neurons	TLR9 agonist (CpG oligonucleotides) induces reduction of cortical and vascular Aβ levels	Improves cognitive function	[84]
	3xTg AD mouse model		TLR9 agonist (CpG ODN) reduces amyloid burden and Tau-related pathology	Reduces amyloid plaque and NFT pathology; cognitive benefit	
**Inflammasome sensors**					
**NLRP1**	Human neurons	Neurons	Link between intraneuronal inflammasome activation, CASP1 activation, and IL-1β-mediated neuroinflammation and CASP6-mediated axonal degeneration	Neuroinflammation, axonal degeneration, and cognitive impairment	[87]
**NLRP3**	Human and mouse brain tissue samples	Microglia and neurons	Mutations associated with familial AD in *Nlrp3*^−/−^ or *Casp*1^−/−^ models showed protection from AD-associated loss of spatial memory and other sequelae	Inflammation, behavioral, and cognitive dysfunction	[88]
	APP/PS1 mouse model		NLRP3 deficiency aligned microglial cells to an M2 phenotype, leading to reduced deposition of Aβ	Elevated brain CASP1 and IL-1β activation, reduced Aβ deposition	
**NLRC4**	Mouse model (C57BL/6 *Nlrc4*^−/−^)	Microglia and astrocytes	LPC induces NLRP3- and NLRC4-dependent inflammasome activation	Astrogliosis, microglial accumulation, alteration in expression of LPC receptor G2A	[89]
			High expression of *NLRC4* in mice astrocytes and human demyelinating-associated disease neurological samples		
**AIM2**	5xFAD mouse model	Microglia	*Aim2* knockout reduces Aβ deposition and microglial activation; no beneficial effect on spatial memory or cytokine expression	Increase in Aβ deposition and microglial activation	[90]
**Other cell surface receptors**					
**TREM2**	5xFAD mouse model	Microglia	TREM2 deficiency enhances Aβ accumulation and neuronal loss	Neurodegeneration	[91]
			TREM2 promotes microglial survival by sustaining microglial response to Aβ plaques and senses anionic lipids interacting with fibrillar Aβ		
	APP/PS1-21 mouse model		TREM2 R47H mutation worsens lipid recognition in AD	Sustaining microglia in response to Aβ accumulation	
**Adaptors**					
**ASC**	Mouse model (C57BL/6 *Asc*^−/−^)	Microglia and astrocytes	Involvement of ASC, CASP1, cathepsin-mediated degradation, calcium mobilization, and potassium efflux in LPC-mediated inflammasome activation	Astrogliosis and microglial accumulation	[89]
**MAVS**	*Agt5*^fl/fl^*Cd11b*^Cre^ mouse model	Microglia	MAVS signaling mediates poly(I:C)-induced inflammation in the brain	Prevents MPTP-induced microglial activation and dopaminergic neuron loss	[92]
			Autophagy negatively regulates the activity of MAVS through direct binding of LC3 to the LIR motif Y(9)xxI(12) of MAVS		
**Other cytosolic sensors**					
**cGAS-STING**	AD patient samples	Microglia	Higher protein levels of STING when compared to control patients; increased phosphorylation of IRF3	Activated pathway affects microglial function	[93]
				Decreased Aβ fibril phagocytosis upon STING’s activation	
**RIG-I**	AD patient samples	Astrocytes	Stimulation with RIG-I increased expression of APP and Aβ	Involved in the pathology of MCI associated with early progression to AD	[94]

**Table 3:** Cell death effector molecules involved in Alzheimer’s disease.

Effectors	Model System	Cell Type	Mechanism	Role in AD Pathogenesis	Reference
**GSDMD**	Mice	Neurons	Aβ_1-42_ induces pyroptosis through GSDMD and NLRP3-CASP1 signaling-mediated GSDMD cleavage	Nerve injury and neuronal loss	[142,145]
			NLRP3/CASP1/GSDMD axis induces neuronal pyroptosis	Increased inflammatory factors (IL-1β and IL-6) in CSF	[146]
**CASP3**	Human and mouse samples	Neurons	CASP3-mediated CASP8, CASP9, and CASP10 processing drive amyloid precursor protein cleavage	Neuronal death and plaque formation	[147]
**CASP6**	AD patient’s CSF samples	NFT	CSF samples indicate TauΔCasp6 level and CASP6 immunoreactivity	Cognitive impairment	[148,149]
			Levels of CSF TauΔCasp6 are inversely correlated with cognitive scores	Tau cleavage	
**CASP7**	Whole genome sequence data of human samples		ADAM10, BACE, and PSEN1/2 secretases process APP	Familial late-onset AD associated with a *CASP7* missense variant	[150]
			Alternative processing of APP results in cleavage of C31 fragment through CASP7		
**CASP8**	Autopsy of brain tissue from hippocampus and entorhinal cortex	Neurons	Activation of apoptotic programs in neurons of AD brain activates death receptor pathway and CASP8	Amyloid processing, synaptic plasticity, learning/memory, controls microglia, proinflammatory activation, and neurotoxicity	[151,152]
			Aβ mediates apoptosis in neurons via Fas/TNF family of death receptors, followed by activation of CASP8 and CASP3		
**CASP9**	Rat PC12 cells; human samples	Neurons	Colocalization of active CASP9 with active CASP8 and accumulation of CASP3-cleavage products of fodrin	Activation in Tau cleavage	[153]
			Activation of CASP9 in neurons positive for oxidative damage to DNA/RNA		
			CASP9 activation leads to NFT formation		
**RIPK1**	Human and rat	Neurons (temporal gyrus tissue)	RIPK1-mediated necroptosis in neuronal cells involves the mTORC1 pathway	Gene expression dysregulations in AD are predicted by RIPK1	[154]
**MLKL**	Human and mice	Neurons (temporal gyrus tissue)	High pMLKL levels and MLKL dimers in AD brains and colocalization of pMLKL with membrane marker cadherin; pMLKL immunoreactivity localized to membrane	Necrosome formation	[154]

**Table 4:** Cytokines and interferons involved in Alzheimer’s disease.

Molecules	Model System	Cell Type	Mechanism	Role in AD Pathogenesis	Reference
**Inflammasome-associated inflammatory cytokines**					
**IL-1α**	TgAPPsw and PSAPP transgenic mice	Brain slices	Increased Aβ	Accumulation of Aβ drives neuroinflammatory responses	[196]
**IL-1β**	In vitro	Microglia	Microglial activation by Aβ	Recruit microglia and astrocytes to Aβ locus	[197]
**IL-18**	AD samples	CSF	TNF-α, IP-10, and IL-18 levels increase linearly with age	Age-related shift from Th1- to non-Th1–related cytokines	[198]
**Pleotropic cytokines**					
**IL-10**	AD samples	CSF	IL-10 correlated with age in a U-shaped relationship	AD accelerates the shift away from Th1 phenotypes	[198]
**Other inflammatory cytokines**					
**IL-6**	−	Microglia	Microglial activation by Aβ	Recruit microglia and astrocytes to Aβ locus	[197]
	TgAPPsw and PSAPP transgenic mice	Brain slices	Increased Aβ	Accumulation of Aβ drives neuroinflammatory responses	[196]
	In vitro	Microglia	Pre-aggregated Aβ_1-42_ exposure to microglia	Increased production of proinflammatory cytokines	[199]
**IL-12**	AD samples and mouse model (SAMP8, APP/PS1)	Microglia	IL-12 inhibition improves AD-like pathology	Ameliorates AD-associated neuropathology and spacial memory; cognitive decline	[143,200,201]
**IL-23**	AD samples and mouse model (SAMP8, APP/PS1)	Microglia	IL-23 inhibition improves AD-like pathology	Ameliorates AD-associated neuropathology and spacial memory; cognitive decline	[143,200,201]
**TNF-α**	AD samples	Neurons	Phase I and IIa clinical trial of TNF-α inhibitors reduces cognitive decline and improves daily activities	Exacerbates Aβ and Tau pathologies	[202]
	−	Microglia	Aβ activates microglia	Recruit microglia and astrocytes to Aβ locus	[197]
	TgAPPsw and PSAPP transgenic mice	Brain slices	Higher levels of Aβ	Accumulation of Aβ drives neuroinflammatory responses	[196]
	In vitro	Microglia	In response to pre-aggregated Aβ_1-42_ treatment, microglia release TNF-α	Increased production of proinflammatory cytokines	[199]
**Interferons**					
**IFN-γ**	−	Neuron, microglia co-culture	Synergistic action of Aβ with IFN-γ or CD40 ligand triggers TNF-α secretion	Production of neurotoxic ROS	[203,204,205]
	TgCRND8 mouse model		AAV-mediated expression of IFN-γ in the brains	Enhanced amyloid plaque clearance; increased astrogliosis and microgliosis; reduced levels of soluble Aβ and Aβ plaque burden	[206]
**Chemokines**					
**MIP-1α**	In vitro	Microglia	Pre-aggregated Aβ_1-42_ exposure to microglia	Increased production of proinflammatory cytokines	[207]
**M-CSF**	In vitro	Microglia	Pre-aggregated Aβ_1-42_ exposure to microglia	Increased production of proinflammatory cytokines	[207,208,209]
	AD samples	Plasma samples	High M-CSF levels	Mild cognitive impairment	
**GM-CSF**	TgAPPsw and PSAPP transgenic mice	Brain slices	Increased Aβ	Accumulation of Aβ drives neuroinflammatory responses	[196]

**Table 5:** Therapeutic options targeting neuro-inflammation and cell death pathways in Alzheimer’s disease.

Target	Therapy/Drug	Mechanism of Action	Impact on AD	Clinical Phase	Reference
**NLRP3**	CP-456,733 (CRID/MCC950)	Diarylsulfonylurea compound specifically inhibits NLRP3 inflammasome	Promotes microglial Aβ clearance; reduces Aβ accumulation; improves cognitive function	Moving to Phase II	[38,125,230,231]
		Reduces cellular release of IL-1β, IL-1α, and IL-18			
	Glyburide	Sulfonylurea-based compound inhibiting NLRP3 inflammasome activation	Mitigates cognitive impairment	−	[230,232]
			Reduces hippocampal neuroinflammation		
**CASP1**	VX-765	Inhibits CASP1, reduces IL-1β/IL-18 release	Prevents progressive Aβ deposition and reverses brain inflammation	−	[38,233]
			Normalizes synaptophysin protein levels		
**GSDMD**	Disulfiram + Bay 11-7082	Inhibits GSDMD-mediated pyroptosis by covalent modification of 191/192 cysteine residue of GSDMD	−	−	
**RIPK1**	DNL747	RIPK1 inhibitor	−	Phase I	[234]
**RAGE**	Azeliragon	Reduces inflammation	Reduces Aβ transport to brain	Phase III	[207]
			Diminishes toxic effects of oligomers		
**TREM2**	AL002	Targets microglial TREM2 receptors	Promotes microglial clearance of Aβ and reduces neurotoxicity	Phase II	[235]
**Neuro-inflammation**	ALZT-OP1 (cromolyn + ibuprofen)	−	Reduces Aβ aggregation.	Phase II	[236,237]
			Induces neuroprotective microglial activation		
	Daratumumab	Targets CD38 on glia cells	Regulates microglial activity	Phase II	[238,239]
	Montelukast	CysLT-1 receptor antagonist	Affects inflammatory processes, neuronal injury, BBB integrity, and Aβ protein accumulation	Phase II	[240,241]
	AL003	Targets SIGLEC-3 (CD33)	Reactivates microglia and brain immune cells	Phase I	[242]
			Aids microglial clearance of toxic proteins		
**TNF-α**	Adalimumab	Humanized anti-TNF-α antibody	Attenuates neuronal damage and neuroinflammation	Preclinical [125]	
			Decreases beta secretase-1 protein expression and Aβ_1-40_ plaques; improves cognitive functions		
	XPro1595	Targets only soluble form of TNF-α	Reduces pre-plaque Aβ pathology and microglia activation	Preclinical [125]	
			Improves synaptic and cognitive functions		
**IL-12/IL-23**	Genetic ablation or pharmacological manipulation	Genetic ablation of IL-12/IL-23 signaling molecules p40, p35, or p19	Reduces cerebral Aβ load	Preclinical [125]	
			Reduces cognitive deficit		
